# Cost-Effectiveness of Extended-Release Methylphenidate in Children and Adolescents with Attention-Deficit/Hyperactivity Disorder Sub-Optimally Treated with Immediate Release Methylphenidate

**DOI:** 10.1371/journal.pone.0127237

**Published:** 2015-05-29

**Authors:** Jurjen van der Schans, Nikos Kotsopoulos, Pieter J. Hoekstra, Eelko Hak, Maarten J. Postma

**Affiliations:** 1 Unit of PharmacoEpidemiology & PharmacoEconomics (PE2), Department of Pharmacy, University of Groningen (RUG), Groningen, the Netherlands; 2 University of Groningen, University Medical Center Groningen, Department of Psychiatry, Groningen, the Netherlands; 3 University of Groningen, University Medical Center Groningen, Institute of Science in Healthy Aging & healthcaRE, Groningen, the Netherlands; Deakin University, AUSTRALIA

## Abstract

**Background:**

Attention-Deficit/Hyperactivity Disorder (ADHD) is a common psychiatric disorder in children and adolescents. Immediate-release methylphenidate (IR-MPH) is the medical treatment of first choice. The necessity to use several IR-MPH tablets per day and associated potential social stigma at school often leads to reduced compliance, sub-optimal treatment, and therefore economic loss. Replacement of IR-MPH with a single-dose extended release (ER-MPH) formulation may improve drug response and economic efficiency.

**Objective:**

To evaluate the cost-effectiveness from a societal perspective of a switch from IR-MPH to ER-MPH in patients who are sub-optimally treated.

**Methods:**

A daily Markov-cycle model covering a time-span of 10 years was developed including four different health states: (1) optimal response, (2) sub-optimal response, (3) discontinued treatment, and (4) natural remission. ER-MPH options included methylphenidate osmotic release oral system (MPH-OROS) and Equasym XL/Medikinet CR. Both direct costs and indirect costs were included in the analysis, and effects were expressed as quality-adjusted life years (QALYs). Univariate, multivariate as well as probabilistic sensitivity analysis were conducted and the main outcomes were incremental cost-effectiveness ratios.

**Results:**

Switching sub-optimally treated patients from IR-MPH to MPH-OROS or Equasym XL/Medikinet CR led to per-patient cost-savings of €4200 and €5400, respectively, over a 10-year treatment span. Sensitivity analysis with plausible variations of input parameters resulted in cost-savings in the vast majority of estimations.

**Conclusions:**

This study lends economic support to switching patients with ADHD with suboptimal response to short-acting IR-MPH to long-acting ER-MPH regimens.

## Background

Attention-Deficit/Hyperactivity Disorder (ADHD) is a common psychiatric disorder, mostly seen and diagnosed in children and adolescents with a prevalence around 6%. [1] Academic failure, poor self-esteem, and troublesome peer and family relationships are associated with ADHD and patients are often diagnosed with one or more co-occurring psychiatric disorders [2]. The majority of diagnosed children and adolescents continue to have impairing symptoms into adulthood [3]. The treatment of ADHD consists of behavioral treatments or pharmacotherapy, alone or in combination [4]. Cost-effectiveness of pharmacotherapy was proven [5] higher when compared to behavioral treatments. Combined pharmacotherapy and behavioral therapy is less cost-effective due to the large increase in costs associated with behavioral treatments [5], although a combination of psychotherapy and pharmacotherapy could be cost-effective in the case of ADHD and comorbid disorders [5].

Psychostimulants present the most commonly used pharmacotherapy. Immediate-release methylphenidate (IR-MPH) is a psycho-stimulant drug indicated for the treatment of ADHD and is the medicine of first choice in most guidelines [6]. Although the methylphenidate has a well-established short term effectiveness in reducing the core symptoms of ADHD compared to placebo treatment, the effectiveness in the long term (>2 years) is still uncertain [7]. In a follow-up of the Multimodal Treatment Study of Children with ADHD (MTA-study), reduced longer term stimulant medication effectiveness was associated with decreasing adherence to the pharmacotherapy [8].

It has been suggested that inconvenience, including the frequent administration, the social stigma in cases of in-school administration and the possibility of drug diversion due to multiple dosings per day may contribute to poor patients’ compliance to IR-MPH [9]. It is estimated that almost 42% of the IR-MPH-treated patients with ADHD are sub-optimally treated due to numerous reasons including reduced adherence [2].

It has been suggested that by replacing a short-acting MPH with a single dosage extended-release formulation, adherence may be improved, which may lead to better health and economic outcomes [10, 11]. Duration of action of extended-release methylphenidate differs among the available products and ranges from 6 to 12 hours, which is considerably longer compared to IR-MPH of which the duration of effect ranges from 3 to 5 hours [12]. Extended-release psycho-stimulants were introduced in the Netherlands in 2003 and since then, their use has been steadily increasing [13]. It has been estimated that in 2006 approximately 30% of all MPH prescriptions were extended-release MPH (ER-MPH) [13]. An earlier cost-effectiveness analysis from our group reported that switching sub-optimally treated youths to long-acting methylphenidate osmotic release oral system (MPH-OROS) was cost-effective, but not cost-saving [2]. In our previous economic analysis, we only included the direct costs of ADHD [2]. However, in a recent review, Le et al., [14] showed that in addition to direct costs, ADHD results in a considerable amount of indirect costs. The aim of the present study was to conduct an updated economic evaluation of the use of ER-MPH in patients who were sub-optimally treated with IR-MPH, and to compare switching to ER-MPH preparations with the continued use of IR-MPH from a societal perspective.

## Methods and Data

### Economic model

**Fig 1:**
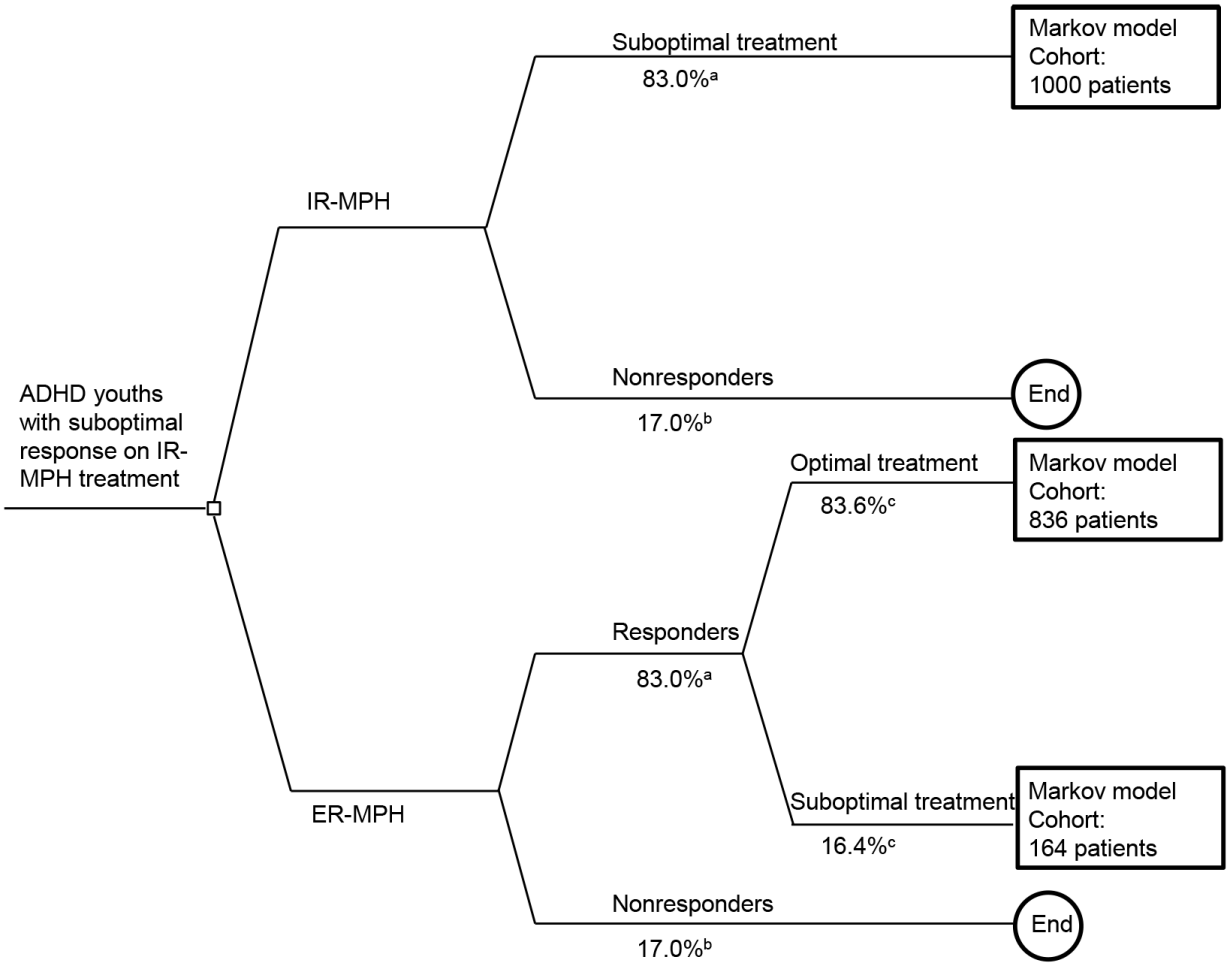
Decision tree of the exploratory initial two-month phase prior to the Markov model. a. Source: Expert panel Faber et al. (2008)[2] b. Source: Expert panel Faber et al. (2008)[2] c. Source: Gau et al. (2008)[17]

The economic model of this study was based on the Markov model reported by Faber et al. [2]. This model was based on (1) assumptions of an expert panel, consisting of three paediatricians and two child psychiatrists, all specialised in ADHD and (2) peer reviewed scientific data. We further refined this previously developed Markov model [15] in which costs and outcomes of a hypothetical cohort of 1,000 patients with ADHD were simulated. For the model, we considered only patients who were sub-optimal responders to IR-MPH treatment due to adherence problems with the multiple doses short-acting regimen (3–5 hours) (see Fig 1). The model assessed the costs and outcomes of sub-optimally treated patients with ADHD switching to ER-MPH, i.e., MPH-OROS, Equasym XL, or Medikinet CR, compared to sub-optimally treated patients with ADHD remaining on IR-MPH. Direct and indirect costs were estimated and outcomes were measured in terms of quality-adjusted life years (QALYs). Equasym XL and Medikinet CR were grouped together because of similar costs/mg in the Netherlands. The model produced incremental cost-effectiveness ratios (ICERs) reported in net costs per QALY. The time horizon of the model was up to 10 years capturing the time interval between the recognition of ADHD at school age (around age 8) until patient’s adulthood; i.e., in the current study potential continuation of ADHD into adulthood was not considered. A societal analytic perspective was selected in order to encompass the wider economic consequences of ADHD to children and their parents, in line with the Dutch guidelines for pharmaco-economic research [16]. According to these same guidelines, all future costs were discounted at 4% and future outcomes at 1.5%.

**Fig 2:**
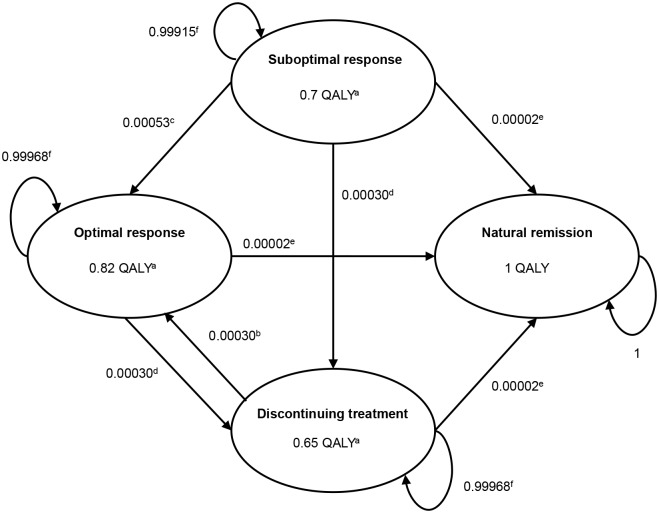
Markov model IR-MPH treatment with daily transition probabilities and utilities on an annual basis (QALY). QALY = Quality adjusted life years a Source: Lloyd et al. (2011) [19] b Source: Wong et al. (2009) [18] c Source: Expert panel Faber et al. (2008) [2] d Source: Faber et al. (2008) [2] e Source: Biederman et al. (2000) [20] f Source: Remaining probabilities

**Fig 3:**
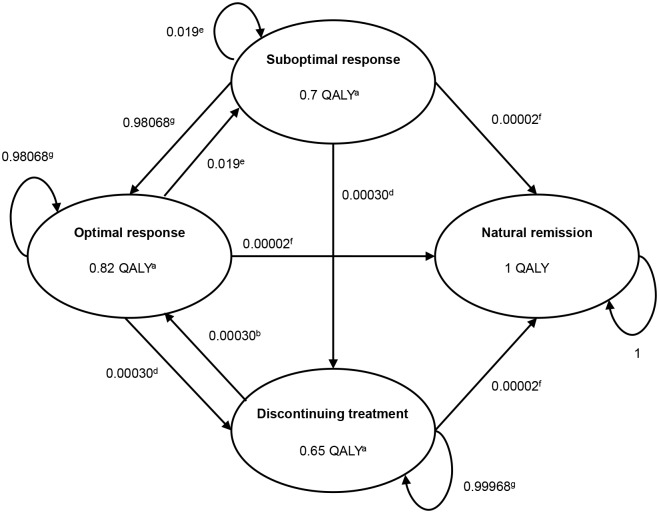
Markov model ER-MPH treatment with daily transition probabilities and utilities on an annual basis (QALY). QALY = Quality adjusted life years a Source: Lloyd et al. (2011) [19] b Source: Wong et al. (2009) [18] c Source: Expert panel Faber et al. (2008) [2] d Source: Faber et al. (2008) [2] e Source: Steele et al. (2006) [21] f Source: Biederman et al. (2000) [20] g Source: Remaining probabilities

An illustration of the economic model developed is presented in Figs 1–3. A decision tree was developed to simulate the first two months of the analysis with sub-optimally treated patients entering the model. The first two-month period was considered the time interval during which a patient was identified either as a true non-responder to MPH (17.0%) [2] or as a potential responder, but with compliance being the essential problem (83.0%). The group of potential responders was subsequently considered to enter the 10-year Markov model (Figs 2–3). Once in the Markov model the IR-MPH and ER-MPH cohorts could progress to four states: (1) staying a sub-optimal responder, i.e. only partial attainable reduction of ADHD symptoms due to ongoing adherence problems, (2) becoming an optimal responder, i.e. maximum attainable reduction of ADHD symptoms given an optimal adherence, (3) discontinuing treatment, i.e. stopping treatment despite the perseverance of ADHD symptoms or (4) going into natural remission, i.e. remission of ADHD symptoms even in the absence of treatment.

The results of an expert panel consultation were used as reported in the Faber et al. study to determine the use of psychosocial interventions, consultations and special education of patients optimally responding to MPH, suboptimal responders and patients who have discontinued treatment.

It was assumed that by switching from IR-MPH to ER-MPH, 83.6% [17] would become an optimal responder, in line with Gau et al. [17] since a European source was not available. Ergo, 16.4% of the patients switching from IR-MPH to ER-MPH were considered not to benefit from the switch [17]. This differed from the original model by Faber et al. [2] where 100% of the patients would become an optimal responder. Each patient could stay in the optimal responder stage, could become a sub-optimal responder due to adherence problems, discontinue the treatment, or go into natural remission. The transition from optimal to suboptimal treatment was not included in the IR-MPH model due to lack of data. As stated, the Markovian transition probabilities used in the model were obtained from the previous economic evaluation of ER-MPH reported by Faber et al. [2] (Figs 2–3). An additional probability of restarting treatment after discontinuation, i.e., the transition between discontinuing treatment to becoming an optimal responder, was investigated to reflect all potential real-life treatment trajectories and outcomes. The probability of restarting treatment in either the IR-MPH or the ER-MPH group was not included in the previous model by Faber et al.

Restarting treatment was defined as a gap of six months or more between prescriptions. In the first year after stopping the treatment, restarting rate was estimated at 11% as derived from Wong et al. [18]. In the second year, a 4% probability was used (derived from the cumulative percentage of 15% probability in two years). In the third and fourth year, an increase of 3% was modelled until the maximum of 18% would be reached [18]. All annual percentages were converted into daily probabilities to be in line with the model structure. In the last six years of the model there was no likelihood of restarting the pharmacological treatment.

All detailed input parameters have been presented in the S1 appendix.

### Base case costs and outcomes

By building on the previously mentioned model and cost estimates used in the Faber et al. study the current economic evaluation study integrated the direct and indirect costs of ADHD with the most recent economic and clinical literature. Direct costs (consultation, intervention, and special education costs) of this study were derived from the economic analysis of Faber et al. [2]. An inflation adjustment was applied to convert the costs to 2013 prices. Pharmacotherapy list prices were obtained from CVZ [22]. In order to quantify the medication costs of each treatment, an epidemiological database research was performed. Information on drug use was obtained from the IADB.nl database, containing pharmacy-dispensing data from community pharmacies in the northern and eastern part of the Netherlands. Dutch patients usually register at a single community pharmacy and therefore this pharmacy can provide an almost complete listing of the subject’s prescribed drugs [23]. The database covers a population of 500,000 people since 1999. A part of the data goes back to 1994. The daily dosing of both the IR-MPH and ER-MPH formulations was determined following the IADB study population (8–18 years old) between January 2007 and December 2011. The average daily dose of the MPH formulations was calculated per age group.

The indirect costs reported by Hakkaart-van Roijen et al. [24] (as recently re-analyzed by Le et al. [14]) were added to incorporate the broader societal economic consequences of ADHD. In the Hakkaart-van Roijen et al. study both the direct and indirect costs of ADHD were reported; however, pharmacotherapy costs were not taken into account. Notably, we only used the indirect costs due to ADHD symptoms from this study and compared these costs to indirect costs made by patients without ADHD. In order to attribute the reported indirect costs to each health state, weighting based on the ratio of direct costs of the different health states, as assessed by the expert panel, was done. First, patients discontinuing treatment were attributed a weight of 100%; next, patients optimally responding to treatment were weighted with 21.7% and patients with a suboptimal response were assigned a weight of 42.9%. The corresponding annual indirect costs due to direct medical costs of the mother and reduced costs due to absenteeism and reduced efficiency at work were €2,517, €546 and €1,081 for patients discontinuing treatment, patients with optimal and sub-optimal response, respectively.

QALYs were calculated from a study reporting utility weights for validated health states based upon trial data and qualitative information from children with ADHD [19]. Thus, the health state descriptions reflected actual patients’ and parents’ experiences. The study derived utility weights for each health state from the general public using the time trade-off (TTO) method, in which the value is determined by the choice of trading length of life for quality of life. The assessment of utility weights from the general public is encouraged by most decision makers such as the UK’s National Institute for Clinical Excellence (NICE) [25]. The utilities per health state are presented in Figs 2–3 as well.

### Scenario and sensitivity analysis

Deterministic univariate sensitivity analysis was performed on all model’s parameters using a variation range of ±25%. In addition, multivariate sensitivity analysis was performed in which the worst-case parameter values for the ER-MPH (with respect to cost-effectiveness) were analysed. The worst-case scenario consisted of a 25% reduction of all costs, combined with a 25% increase in the costs of the ER-MPH formulations, a reduction of 25% of use of consultation, interventions, and special education in the suboptimal response and discontinuing treatment state, a 25% increase in transition probability of suboptimal response to optimal response in the IR-MPH model, and a 25% increase in transition probability of optimal response to suboptimal response and discontinuing treatment in the ER-MPH model. Furthermore, in order to assess the variation of effectiveness of switching from IR-MPH to ER-MPH, a scenario was run considering the non-effective group of switchers as being re-starters. A probabilistic sensitivity analysis (PSA) was also performed by assigning probability distributions to the different parameters of the model. The distributions of the original studies were used for the effects and transition probabilities, if available. Cost ranges were identified using the expert panel [2]. Common distributions used in a probabilistic sensitivity analyses of similar cost-effectiveness studies were used [26]. The gamma distribution was used for the costs and utilities. The beta distribution was used for the transition probabilities and the normal distributions for the expert panel consultation results. The results of the PSA were summarised in a cost effectiveness plane. In the PSA an overlap of the QALY distributions may result in suboptimal treatment having more effect than optimal treatment. Although in a few cases this may be possible, we assumed that this was not possible for the vast majority of the cohort. Also, whenever this would be the case in real life, it would rather have resulted from occurrences beyond the types modelled in our design. Therefore, the assumption of a fixed sequence was used. Whenever the sequence of QALYs did not match the sequence: “optimal treatment outcomes > suboptimal treatment outcomes > treatment discontinuation”, the simulation was replaced. By assuming a fixed sequence we implied that treating a patient optimally is always better than suboptimal treatment and suboptimal treatment is better than treatment discontinuation, which seems a logical sequence within the approach from a pure pharmaceutical perspective being chosen.

## Results

### Base-case cost effectiveness

Table 1 illustrates the costs and consequences of the analyses and the 95%-confidence intervals (95%CI). Notably, both long-acting treatment options investigated were estimated to render cost savings of €4200 to 5400 per patient over the 10-year period investigated.

**Table 1:** Markov model results of base case analysis per switcher per 10 years.

Costs and outcomes	IR-MPH	MPH-OROS	Medikinit CR/ Equasym XL
Medication costs	€ 769 (€573-€966)	€ 3,187 (€ 2,248-€ 4,144)	€ 1,977 (€1,425-€2,516)
Consultation costs	€ 4,496 (€3,413-€5,312)	€ 3,854 (€2,897-€4,685)	€ 3,854 (€2,897-€4,685)
Intervention costs	€ 5,228 (€4,233-€5,966)	€ 4,087 (€3,236-€4,752)	€ 4,087 (€3,236-€4,752)
Special education	€ 6,547 (€4,282-€8,952)	€ 4,740 (€2,852-€6,768)	€ 4,740 (€2,852-€6,768)
Indirect costs	€ 9,404 (€6,852-€11,683)	€ 6,267 (€3,921-€8,383)	€ 6,267 (€3,921-€8,383)
Non-responder costs	€ 22(€18-€25)	€ 93 (€70-€112)	€ 62 (€48-€75)
Total costs	€ 26,464(€20,897-€31,172)	€ 22,229 (€17,191-€26,432)	€ 20,988 (€16,155-€25,151)
Total QALYs	6.855(4.852-8.613)	7.173 (5.253-8.830)	7.173 (5.253-8.830)
Costs per QALY	€ 3,861 (€2,714-€5,562)	€ 3,099 (€2,089-€4,366)	€ 2,926 (€2,848-€3,075)
Cost difference vs. IR-MPH		-€ 4,235 (€-2,904- €-5,335)	-€ 5,477 (€-4,394- €-6,268)
QALYs difference vs. IR-MPH		0.318 (0.053-1.285)	0.318 (0.061-1.313)

IR-MPH = Immediate-release methylphenidate

MPH-OROS = Methylphenidate osmotic release oral system

QALY = Quality adjusted life years

Compared to the IR-MPH, MPH-OROS resulted in cost savings and QALY gains. Hence, the results suggest that MPH-OROS is a cost-effective option in patients sub-optimally treated with IR-MPH who switch to MPH-OROS. The comparison of MPH-Equasym XL/Medikinet CR with IR-MPH produced a better result, again showing cost-effectiveness of long-acting pharmacotherapies over IR-MPH. The incremental cost-effectiveness ratio vs. IR-MPH is therefore dominant for both MPH-OROS and Medikinit CR/Equasym XL given that the intervention is cost-saving with a net positive health gain.

The resulting amounts of cost-savings were mainly explained by the differences in costs of medication and by differences in indirect costs, special education costs, consultation costs and intervention costs. Medication costs in the MPH-OROS and Equasym XL/Medikinet CR long-acting pharmacotherapies were up to four times higher than for IR-MPH. Yet, indirect costs, consultation costs and special education costs accrued to higher total costs for IR-MPH.

Without the indirect costs, responsible for most of the difference between the IR-MPH and ER-MPH scenarios, the difference between both treatments was still cost saving in favor of the ER-MPH treatment (MPH-OROS € -1,099, 95%CI: € -2,071-€ 98).

The base case results suggested that substantial cost-savings could be produced by switching sub-optimally treated patients with IR-MPH to long-acting pharmacotherapy. Assuming an ADHD population medicated with IR-MPH of 36,000 in a population of 3 million children and adolescents in the Netherlands [13, 27], it is estimated that savings of up to €67 million may accrue over a 10-year period (when assuming a 42% suboptimal response population of IR-MPH treated patients with ADHD and 83% MPH responders (see Fig 1)) (= 36,000 * 42% * 83% * costs saved/patient/10 years).

### Scenario and sensitivity analysis

The univariate sensitivity analysis showed that in the vast majority of estimations, switching patients with ADHD who are sub-optimally treated with IR-MPH to a long-acting pharmacotherapy saved costs and was more effective. The parameter which appeared to alter these results was the percentage of patients benefiting from switching from IR-MPH to one of the available ER-MPH regimens. Yet, even when lowering this percentage from 83.6% to 41%, the ICER still indicated cost-effectiveness and cost-saving for all long-acting pharmacotherapies.

Also in the worst case scenario (see methods) for the ER-MPH treatment, all ER-MPH pharmacotherapies remained cheaper and more effective. Cost-savings of € 519 and € 257 were estimated for MPH-OROS and the other two available long-acting pharmacotherapies, respectively, compared to the IR-MPH treatment. All ER-MPH treatments still resulted in QALY gains of 0.08 compared to the IR-MPH treatment.

**Fig 4:**
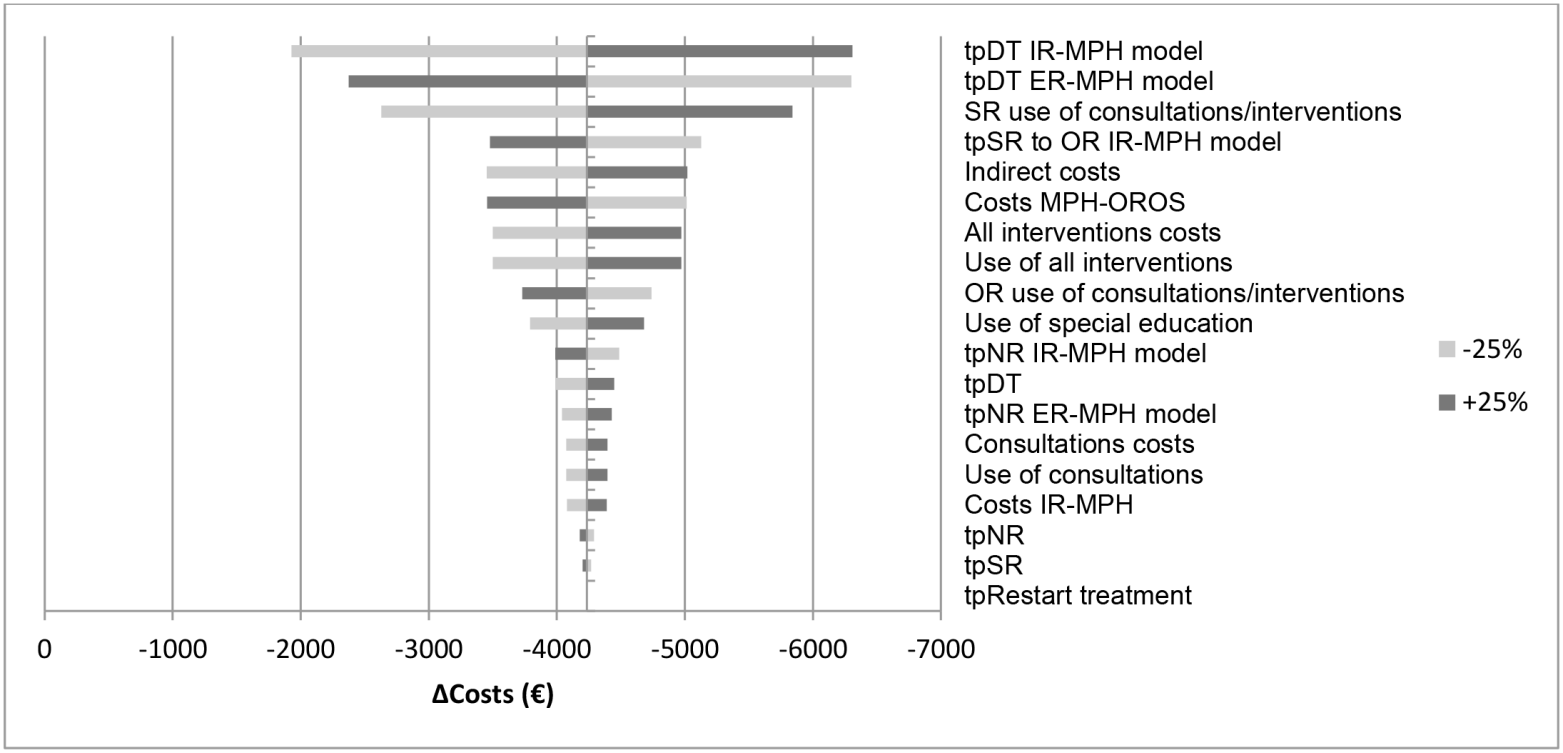
Effect of the univariate sensitivity analyses on the outcome of costs (€) (IR-MPH vs ER-MPH). Illustrative result for ER-MPH, example of MPH-OROS. tp = transition probability, SR = suboptimal response, OR = optimal response, DT = discontinuing treatment, NR = natural remission, IR-MPH = immediate release methylphenidate, ER-MPH = extended release methylphenidate.

**Fig 5:**
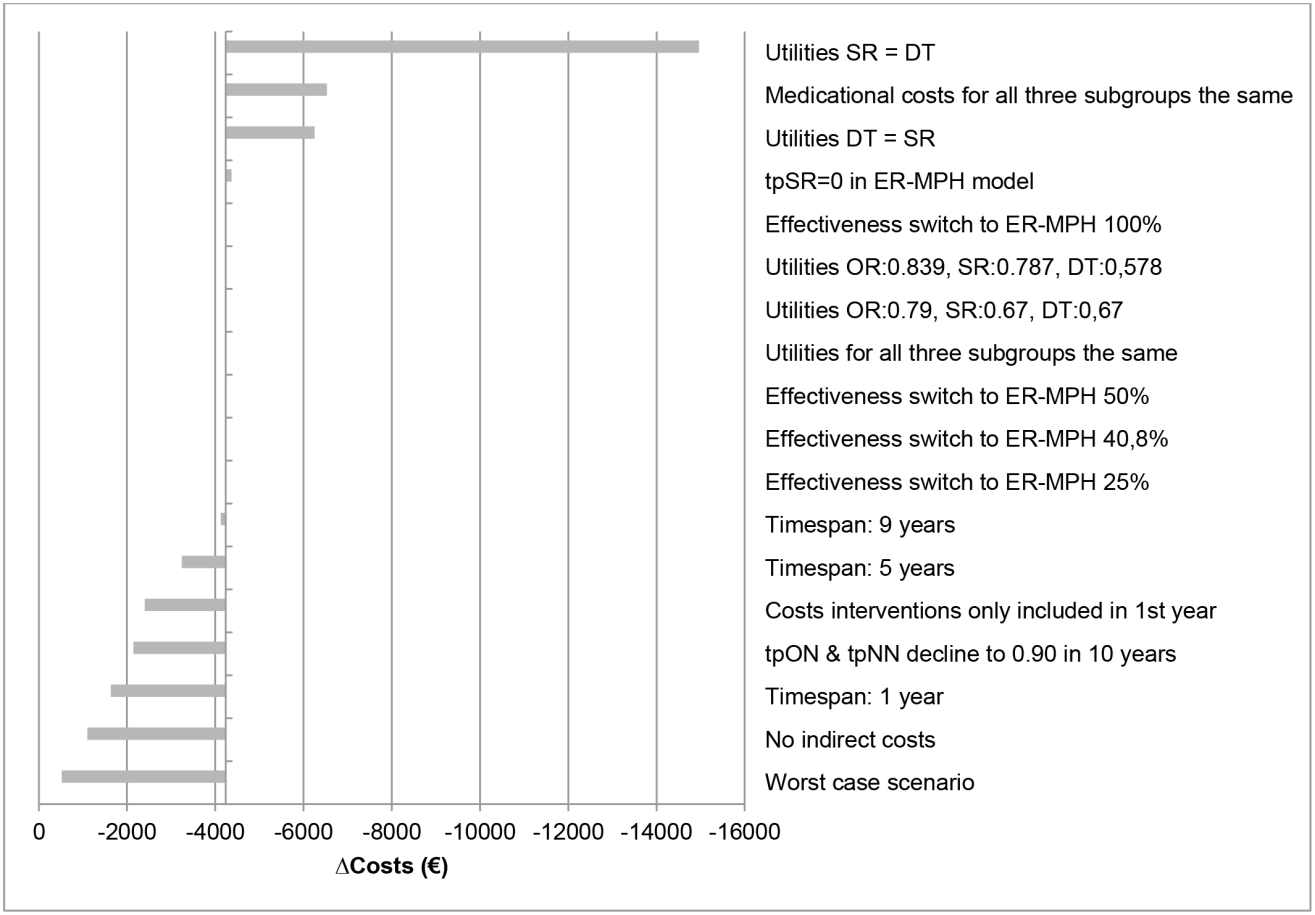
Effect of the scenario analyses on the outcome of costs (€) (IR-MPH vs ER-MPH). Illustrative result for ER-MPH, example of MPH-OROS. tp = transition probability, SR = suboptimal response, OR = optimal response, DT = discontinuing treatment, NR = natural remission, IR-MPH = immediate release methylphenidate, ER-MPH = extended release methylphenidate.

**Fig 6:**
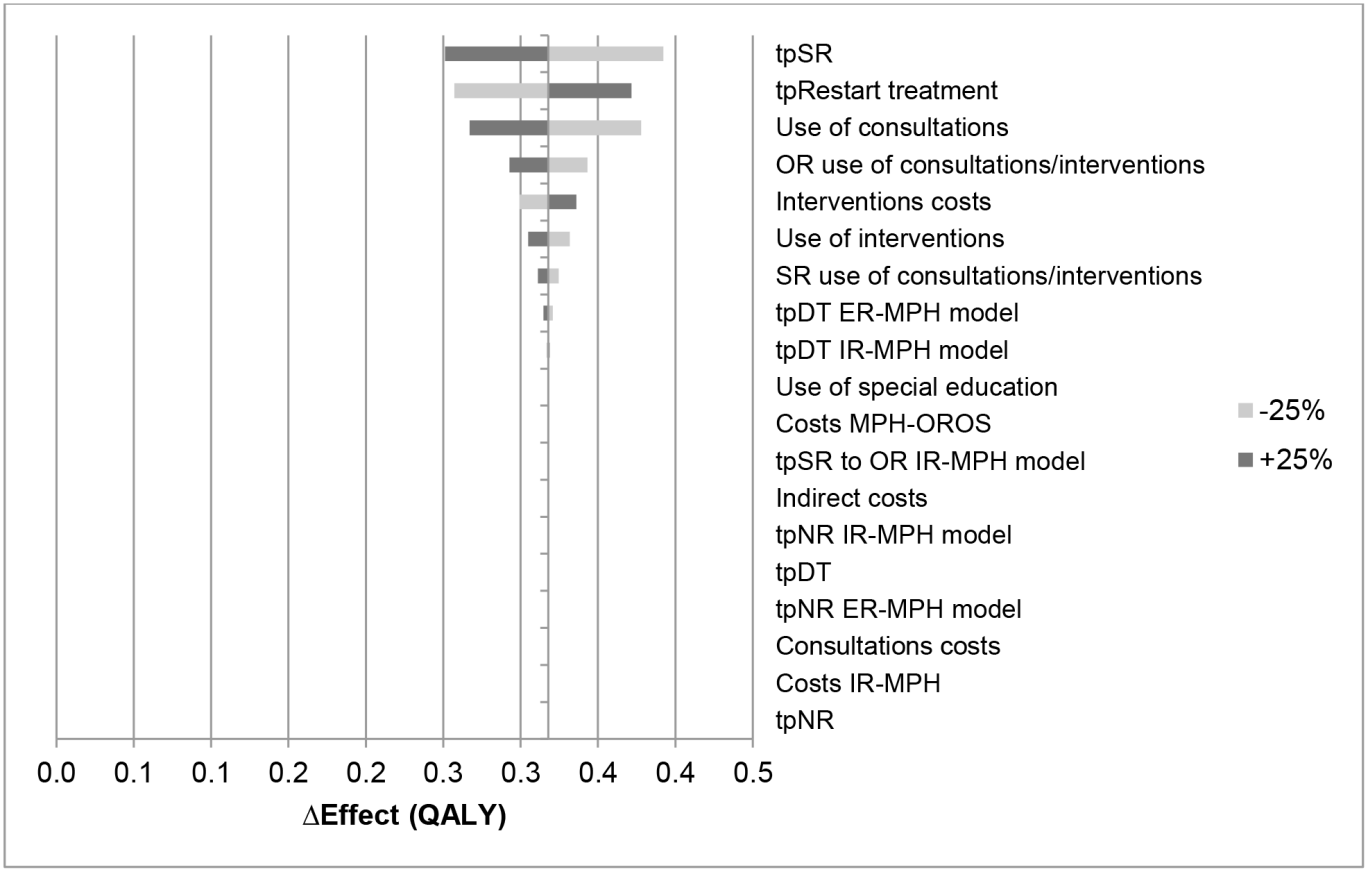
Effect of the univariate sensitivity analyses on the outcome of effect (QALY) (IR-MPH vs ER-MPH). Illustrative result for ER-MPH, example of MPH-OROS. tp = transition probability, SR = suboptimal response, OR = optimal response, DT = discontinuing treatment, NR = natural remission, IR-MPH = immediate release methylphenidate, ER-MPH = extended release methylphenidate.

**Fig 7:**
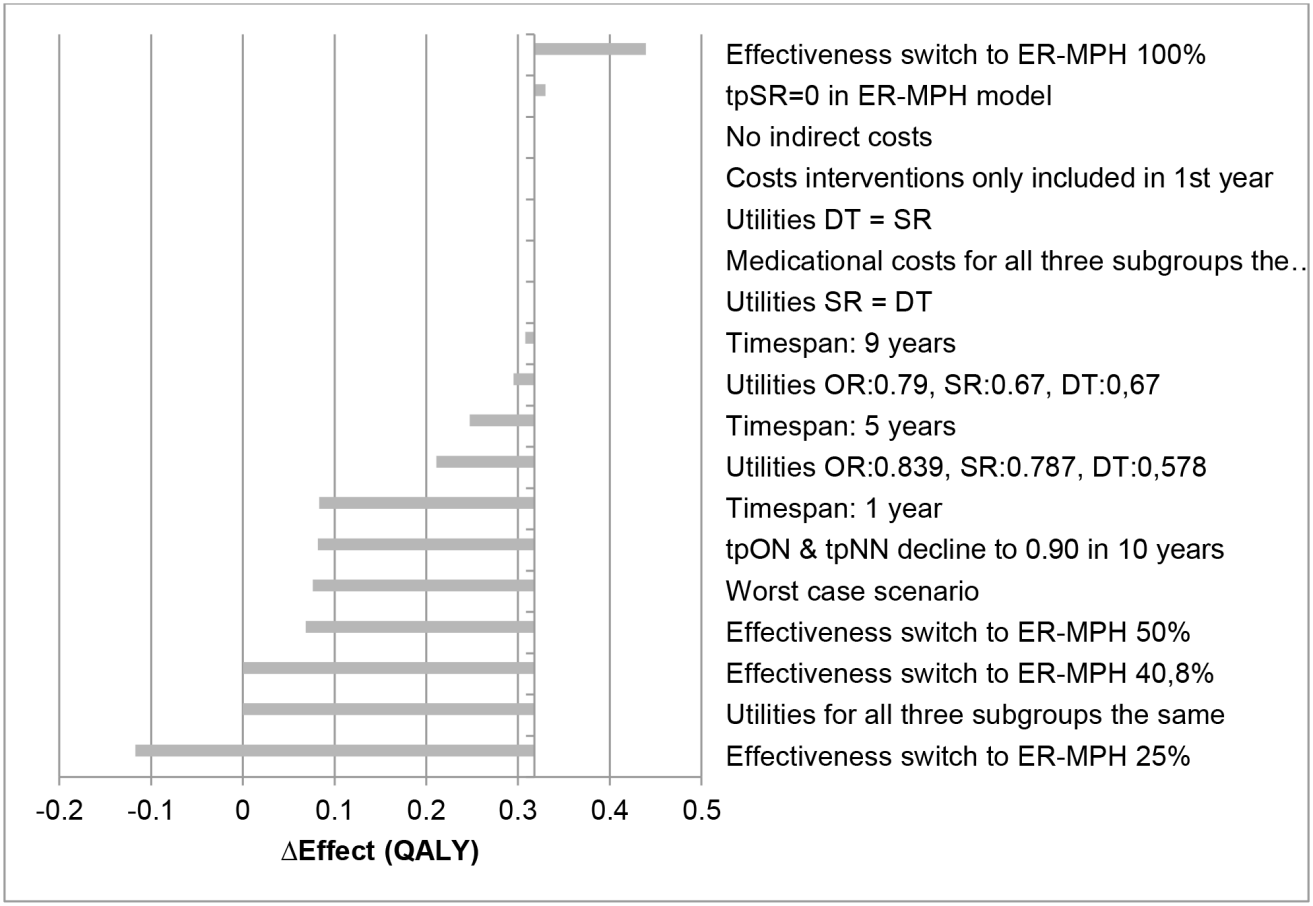
Effect of the scenario analyses on the outcome of effect (QALY) (IR-MPH vs ER-MPH). Illustrative result for ER-MPH, example of MPH-OROS. tp = transition probability, SR = suboptimal response, OR = optimal response, DT = discontinuing treatment, NR = natural remission, IR-MPH = immediate release methylphenidate, ER-MPH = extended release methylphenidate.

**Fig 8:**
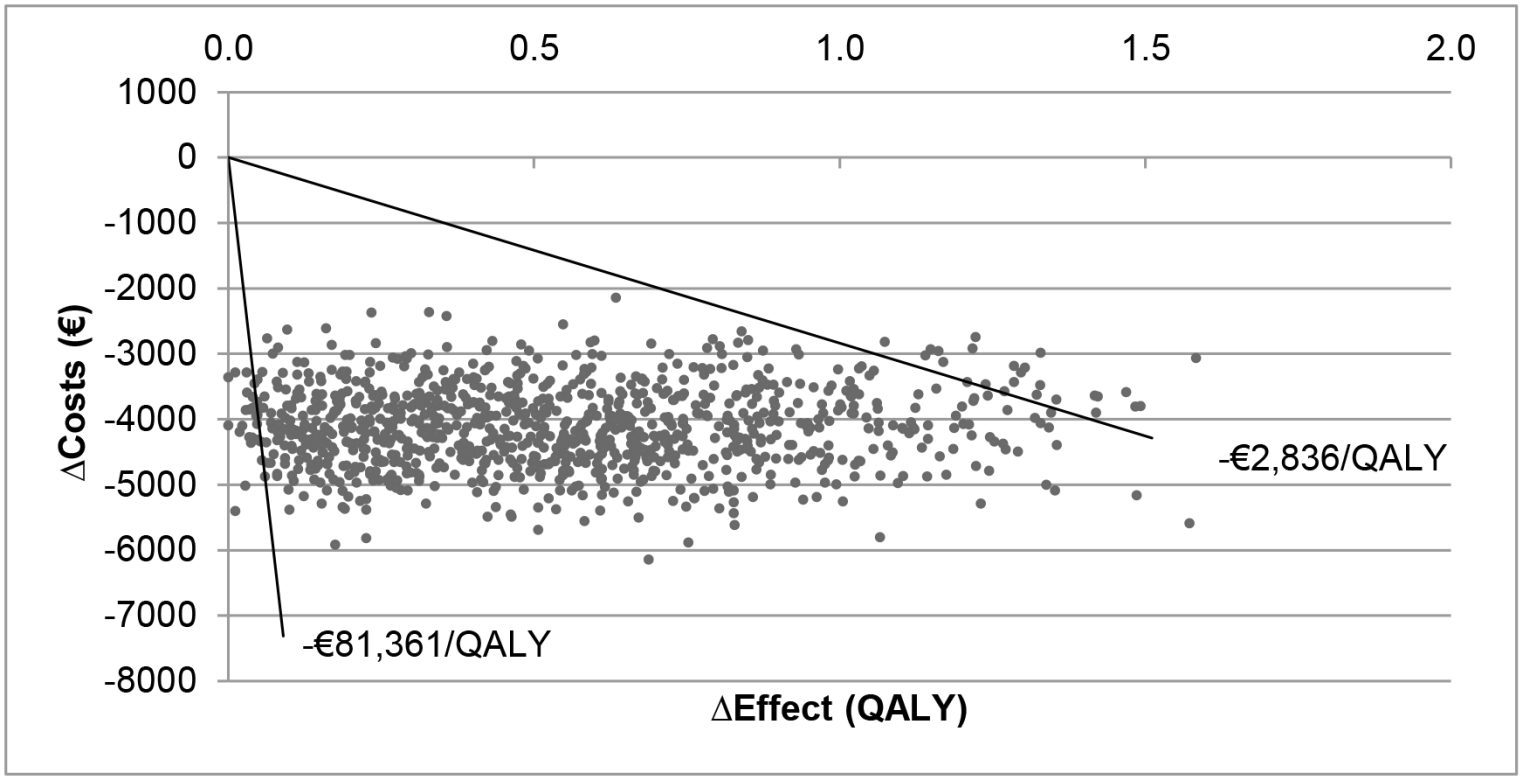
Cost and effect differences of probabilistic sensitivity analysis in cost-effectiveness plane and 95% confidence interval (Illustrative result for ER-MPH, example of MPH-OROS). QALY = Quality adjusted life years

The results of the sensitivity analyses are given in Figs 4–7 and presented as the difference in costs and effect compared to the IR-MPH group. Figs 4 and 6 show the effect on costs and effects, respectively, when varying the different parameters with 25%. Figs 5 and 7 show the effect on costs and effects respectively when applying different scenarios to the model. The results of the probabilistic sensitivity analysis are presented in Fig 8 for MPH-OROS, with again very similar results for two ER-MPH products that we analysed. The results of both the univariate and probabilistic sensitivity analyses of Equasym XL/ Medikinet CR have been presented in the S2 appendix.

The probabilistic sensitivity analysis of the other two ER-MPH treatments was similar to the MPH-OROS treatment except for the higher cost-savings compared to the base case due to the lower pricing of the products. The 90% confidence box shows a differential effect ranging from 0.063 to 1.287 QALYs. The cost difference ranged from € -4,402 to € -6,267.

## Discussion

This cost-effectiveness analysis in children and adolescents with ADHD shows that switching sub-optimally treated patients from short-acting IR-MPH to long-acting ER-MPH is highly likely to result in societal cost-savings while increasing quality of life. Our results were mainly influenced by the actual price of the products on one hand and the estimated indirect cost savings and lowered special education costs on the other hand.

Considering the fact that a generic form of the MPH-OROS has entered the market has significantly improved the cost-effectiveness of ER-MPH compared to the short acting MPH.

When varying the parameters of the model the results suggested that ER-MPH remained a cost-saving option in the vast majority of the scenarios analysed. The parameter which could change the direction of the results is the degree of patients for whom switching from IR-MPH to ER-MPH is beneficial. The threshold analysis showed that if the percentage of patients benefiting from the switch is below 40.8%, less QALYs are produced, whereas cost savings still remain. Although there is scarce evidence showing the benefit of switching sub-optimally treated patients from IR-MPH to ER-MPH, it seems unlikely that this relatively low threshold will be reached considering the sub-optimal state is caused by the daily multiple dose regimen.

The database research showed that medication doses of the long-acting MPH were higher than the short-acting variants. This is a trend also seen in the OBSEER study [28] and may be responsible for the better treatment response. Increasing dosing of IR-MPH as an alternative to switching to ER-MPH will likely only increase the non-compliance of patients and thus may in the end be less effective than the higher dosing long-acting MPH.

A limitation of the used model was that we did not take into account the fact that ADHD is often a co-morbid disorder, and other diseases such as psychiatric disorders [29] but also atopic diseases may be present as well [30]. Suboptimal treatment due to co-morbid disorders could cause an overestimation of the adherence problems and thus overestimate the effect of switching to ER-MPH. Also the possibility of diversion and abuse was not considered in this model although it is expected that single-dose ER-MPH regimens are less likely to be misused [31]. Furthermore, the utilities used in our model differ from the utility weights used by Faber et al.[2]. The latter study did not take the general public’s preferences into account and health states were assessed based on parental rating [32]. In this respect, we would argue that our analysis adheres to the most recent insights into this topic. Finally, our model was based on assumptions of an expert panel and not on real data. Although our sensitivity analyse have indicated the robustness of the model outcome under different scenarios, ultimately it should be acknowledged that the assumptions of the expert panel have been subjective.

The transition from optimal treatment to suboptimal treatment is not possible in the IR-MPH treatment pathway because of a lack of data on this matter. When including this transition this would be in favour of the ER-MPH treatment because of a higher amount of suboptimally treated patients in the IR-MPH treatment pathway. Thus, we would argue that despite this lack in the model, the model tends to estimate savings and QALY gains conservatively, according to common practice in pharmacoeconomic research.

Transition probabilities of being non-compliant in the ER-MPH group is lacking for longer follow-up times. It was assumed that the 8-week compliance was similar to the 10- year model compliance but it is likely that the compliance will worsen over the timespan of 10 years. However, our sensitivity analysis showed that with a linear decline to a daily non-compliance probability of 0.9 in the tenth year our model was still cost saving while QALYs were gained compared to IR-MPH treatment.

Also, the model is restricted to an up to 10-year timespan until adulthood is reached. Although it is uncertain what the effects will be of switching to ER-MPH into adulthood, it is likely that there will not be a negative effect compared to IR-MPH and a longer timespan will only strengthen the effect of switching to ER-MPH. Despite ADHD being a chronic disorder, the base case of the ADHD treatment duration could also be less than the assumed 10 years due to the fact that the effectiveness and adherence after 2 years is still uncertain [8] and many patients in clinical practice do not [13] and should not receive medical treatment for such a long time span. Although the 9, 5 and 1 year timespan of the model in the scenario analysis show a reduction in costs savings and effect gaining, the main outcome of the model remains cost-effective compared to the IR-MPH treatment.

More extensive research into (non-)compliance in ER-MPH treatment will help to further improve this aspect in our model. A final limitation relates to the general ‘memory-less’ feature of a Markov modelling approach. Hence, patients with ADHD, who discontinued treatment, restart treatment in the optimal group but are more likely to stop treatment again or be non-compliant, cannot explicitly be taken into account in this study, whereas in real-life practice it can actually lead to lower effectiveness of switching. This may also apply to the sub-optimally treated patients who are non-compliant to the treatment. Future research should focus on collecting pragmatic evidence on the reasons of poor compliance in ADHD, on presence of co-morbid conditions that may also benefit from beneficial ADHD treatment, and evidence on the effectiveness of switching to ER-MPH.

## Conclusions

This study showed that the long acting ER-MPH results in costs up to four times higher than the short acting IR-MPH. Despite the higher costs, the longer-term economic analysis suggests that switching sub-optimally treated patients from IR-MPH to ER-MPH is very likely to be cost-saving and more effective.

The results of this analysis suggest that over a period of 10 years, considerable cost-savings may be produced for the society and the health-care system. Further research is needed to quantify, based on real-life data, the extent of compliance problems with IR-MPH and the effectiveness of switching to ER-MPH. Such evidence will inform the measurement of the economic impact on both national and European health budgets.

## Supporting Information

S1 Appendix Supporting Appendix. (DOCX)

S2 Appendix Supporting Appendix. (DOCX)
