# Supplementary material for: Cost-Effectiveness of Extended-Release Methylphenidate in Children and Adolescents with Attention-Deficit/Hyperactivity Disorder Sub-Optimally Treated with Immediate Release Methylphenidate
Source: PLoS One. 2015 May 29;10(5):e0127237. doi: 10.1371/journal.pone.0127237 (PMC4449164; doi:10.1371/journal.pone.0127237)
Supplement: S1 Appendix — (DOCX) [file pone.0127237.s001.docx]

## S1 Appendix. Detailed input parameters of the cost-effectiveness model.

**Table A.** Mean percentage and duration as reported by Faber et al. (2008) [2] including standard error (SE) of interventions and special education per year for the different health states, determined by the expert panel.

| **Types of interventions and special education** | Optimal response | | | | | Suboptimal response | | | | | Treatment stopped | | | | |
| --- | --- | --- | --- | --- | --- | --- | --- | --- | --- | --- | --- | --- | --- | --- | --- |
|  | % | SE | No. of contacts | | SE | % | SE | No. of contacts | | SE | % | SE | No. of contacts | | SE |
| **Youths aged ≤ 12 years** | | | | | | | | | | | | | | | |
| Interventions: | | | | | | | | | | | | | | | |
| psycho-education | 89.4 | 2.28 | 2.6 | | 0.07 | 92.9 | 2.37 | 3.6 | | 0.09 | 100 | 2.55 | 3.9 | | 0.10 |
| parent training | 48.5 | 1.24 | 8.3 | | 0.21 | 75.8 | 1.93 | 7.9 | | 0.20 | 78.6 | 2.01 | 14 | | 0.36 |
| behaviour therapy | 7.1 | 0.18 | 13.2 | | 0.34 | 22.8 | 0.58 | 11.8 | | 0.30 | 56.9 | 1.45 | 13.2 | | 0.34 |
| social skills | 18.5 | 0.47 | 9.2 | | 0.23 | 28.5 | 0.73 | 9.8 | | 0.25 | 38.3 | 0.98 | 9.2 | | 0.23 |
| teacher training | 43.4 | 1.11 | 1.8 | | 0.05 | 57.1 | 1.46 | 3.7 | | 0.09 | 65.6 | 1.67 | 3.9 | | 0.10 |
| remidial teaching | 37.1 | 0.95 | 20 | | 0.51 | 50.8 | 1.30 | 20 | | 0.51 | 77.1 | 1.97 | 20 | | 0.51 |
| home training | 4.3 | 0.11 | 10 | | 0.26 | 12.9 | 0.33 | 11.2 | | 0.29 | 32.8 | 0.84 | 14.3 | | 0.36 |
| outpatient | 0 | 0 | 0 | | 0 | 0 | 0 | 0 | | 0 | 25.1 | 0.64 | 51.8 days | | 1.32 |
| institutionalization | 0 | 0 | 0 | | 0 | 0 | 0 | 0 | | 0 | 2.6 | 0.07 | 90 days | | 2.30 |
| Special education: | 1.5 | 0.04 | 0 | | 0 | 12.2 | 0.31 | 0 | | 0 | 43.6 | 1.11 | 0 | | 0 |
|  | | | | | | | | | | | | | | | |
| **Youths aged ≥ 12 years** | | | | | | | | | | | | | | | |
| Interventions: | | | | | | | | | | | | | | | |
| psycho-education | 94.3 | 2.41 | 2.8 | 0.07 | | 90.1 | 2.30 | 3.6 | 0.09 | | 89.4 | 2.28 | 5.4 | 0.14 | |
| parent training | 30.6 | 0.78 | 5.9 | 0.15 | | 44.2 | 1.13 | 8.2 | 0.21 | | 74.3 | 1.90 | 13.7 | 0.35 | |
| behaviour therapy | 9.3 | 0.24 | 10 | 0.26 | | 27.7 | 0.71 | 11.4 | 0.29 | | 56.3 | 1.44 | 12.9 | 0.33 | |
| social skills | 7.1 | 0.18 | 9.2 | 0.23 | | 26.3 | 0.67 | 11.4 | 0.29 | | 52.8 | 1.35 | 10.6 | 0.27 | |
| teacher training | 10 | 0.26 | 2 | 0.05 | | 32.8 | 0.84 | 2.5 | 0.06 | | 31.6 | 0.81 | 3.7 | 0.09 | |
| remidial teaching | 1.5 | 0.04 | 20 | 0.51 | | 38.6 | 0.98 | 20 | 0.51 | | 47.3 | 1.21 | 20 | 0.51 | |
| home training | 0 | 0 | 0 | 0 | | 10.1 | 0.26 | 10 | 0.26 | | 13.2 | 0.34 | 10.1 | 0.26 | |
| outpatient | 0 | 0 | 0 | 0 | | 0 | 0 | 0 | 0 | | 26.5 | 0.68 | 51.8 days | 1.32 | |
| institutionalization | 0 | 0 | 0 | 0 | | 0 | 0 | 0 | 0 | | 4 | 0.10 | 135 days | 3.44 | |
| Special education: | 0.1 | 0.00 | 0 | 0 | | 8.6 | 0.22 | 0 | 0 | | 37.1 | 0.95 | 0 | 0 | |
|  | | | | | | | | | | | | | | | |
| Utilization costs of nonpharmacological interventions were applied once for each age group (once in year 1 for youths aged ≤12 years and once in year 6 for youths aged >12 years). | | | | | | | | | | | | | | | |

## Table B. Mean number as reported by Faber et al. (2008) [2] and standard error (SE) of consultations per year for the different health states, determined by the expert panel.

| **Consultations** | Optimal response | SE | Suboptimal response | SE | Treatment stopped | SE |
| --- | --- | --- | --- | --- | --- | --- |
| **Youths aged ≤ 12 years** | | | | | | |
| Specialist | 2.3 | 0.06 | 3.4 | 0.09 | 6.4 | 0.16 |
| General practitioner | 0 | 0 | 0 | 0 | 0.6 | 0.02 |
| Crisis contact | 0.6 | 0.02 | 1.5 | 0.04 | 2.7 | 0.07 |
|  | | | | | | |
| **Youths aged ≥ 12 years** | | | | | | |
| Specialist | 2.4 | 0.06 | 3.6 | 0.09 | 5.1 | 0.13 |
| General practitioner | 0 | 0 | 0.3 | 0.01 | 0.4 | 0.01 |
| Crisis contact | 0.4 | 0.01 | 1.3 | 0.03 | 3.0 | 0.08 |

##

**Table C**. Mean (standard error) of transition probabilities between health states of the Markov model based on the study of Faber et al. (2008) [2].

| To → From ↓ | Optimal response | | Suboptimal response | | Treatment stopped | Functional remission | |
| --- | --- | --- | --- | --- | --- | --- | --- |
| **Immediate release methylphenidate pathway** | | | | | | | |
| Optimal respons | | 0,99968^a^ | | 0^b^ | 0,00030(0,0008)^c^ | | 2,28311E-05(6,03E-6)^d^ |
| Suboptimal respons | | 0,00053^e^ | | 0,99915^a^ | 0,00030(0,0008)^c^ | | 2,28311E-05(6,03E-6)^d^ |
| Treatment stopped | | 0,00030(0,00004)^g^ | | 0^b^ | 0,99968^a^ | | 2,28311E-05(6,03E-6)^d^ |
| Functional remission | | 0 | | 0^b^ | 0 | | 1 |
| **Extended release methylphenidate pathway** | | | | | | | |
| Optimal respons | | 0,98068^a^ | | 0,019(0,002)^h^ | 0,00030(0,0008)^c^ | | 2,28311E-05(6,03E-6)^d^ |
| Suboptimal respons | | 0,98068^a^ | | 0,019(0,002)^h^ | 0,00030(0,0008)^c^ | | 2,28311E-05(6,03E-6)^d^ |
| Treatment stopped | | 0,00030(0,00004)^g^ | | 0^f^ | 0,99968^a^ | | 2,28311E-05(6,03E-6)^d^ |
| Functional remission | | 0 | | 0^f^ | 0 | | 1 |
| a. These probabilities are 1 minus the remainder in the row.  b. Not applicable in the IR-methylphenidate pathway.  c. Source Database, Day 0-180 0,0003, after day 180 0,0002  d. Source Bierderman et al. (2000) [32] 0,1/(12*365)  e. Source expert panel of Faber et al. (2008) [2]. A probability of 0,00053 in the first year, following year a linear decline until 0 in the 10th year.  f. Not applicable in the extended release methylphenidate pathway.  g. Source Wong et al. (2009) [18] year 1 0,0003, year 2 0,00011, year 3&4 0,00004  h. Source Steele et al. (2006) [31] | | | | | | | |

**Table D.** Quality-adjusted life year (QALY) mean and standard error (SE) of different health states as presented by Lloyd et al (2011) [22].

| **Treatment states** | **QALY** | **Standard  error** |
| --- | --- | --- |
| Suboptimal treated | 0.70 | 0.20 |
| Optimal treated | 0.82 | 0.19 |
| Treatment stopped | 0.65 | 0.21 |
| Functional remission | 1 | - |

**Table E.** Mean costs and standard error (SE) per unit, converted to 2013 prices, used in the cost-effectiveness model.

| **Medication costs [19]** | | | | | | |
| --- | --- | --- | --- | --- | --- | --- |
|  | Mean costs | SE | |  | |  |
| IR methylphenidate | € 0.013 | € 0.002 | | per mg | | Based on 10 mg tablet |
| Methylphenidate OROS | € 0.047 | € 0.006 | | per mg | | based on 36 mg tablet |
| Equasym XL/ Medikinet CR | € 0.040 | € 0.010 | | per mg | | Based on 40 mg tablet |
| Pharmacy fee^a^ | € 7.84 | € 1.00 | | per prescription | |  |
| **Consultation costs^a^** | | | | | | |
| Specialist | € 95.61 | € 12.20 | | per contact | | |
| GP | € 19.05 | € 2.43 | | per contact (>20 min.) | | |
| Crisis contact | € 127.78 | € 16.30 | | per contact (0-99 min.) | | |
| **Intervention costs [2]** | | | | | | |
| Psycho education | € 109.73 | | € 14.00 | | per contact | |
| Parent training | € 102.79 | | € 13.11 | | per contact | |
| Behavioral training | € 109.73 | | € 14.00 | | per contact | |
| Social skill training | € 109.73 | | € 14.00 | | per contact | |
| Teacher training | € 75.08 | | € 9.58 | | per contact | |
| Remedial teaching | € 57.75 | | € 7.37 | | per contact | |
| Home training | € 113.04 | | € 14.42 | | per contact | |
| Outpatient treatment | € 150.50 | | € 19.20 | | per contact | |
| Institutionalization | € 300.99 | | € 38.39 | | per contact | |
| Special education | € 13.92 | | € 1.77 | | per contact | |
| **Indirect costs [21]** | | | | | | |
| Treatment stopped | € 6.90 | | € 0.88 | | per year/patient | |
| Suboptimal treated | € 2.95 | | € 0.38 | | per year/patient | |
| Optimal treated | € 1.50 | | € 0.19 | | per year/patient | |
| a. Costs determined by the Dutch Healthcare Authority in 2012 (www.nza.nl) | | | | | | |
