# Supplementary material for: Cost-Effectiveness of Extended-Release Methylphenidate in Children and Adolescents with Attention-Deficit/Hyperactivity Disorder Sub-Optimally Treated with Immediate Release Methylphenidate
Source: PLoS One. 2015 May 29;10(5):e0127237. doi: 10.1371/journal.pone.0127237 (PMC4449164; doi:10.1371/journal.pone.0127237)
Supplement: S2 Appendix — (DOCX) [file pone.0127237.s002.docx]

## S2 Appendix. The results of the univariate and probabilistic sensitivity analyses of Equasym XL/ Medikinet CR.

## Figure A. Effect of the univariate sensitivity analyses on the outcome of costs (€) (IR-MPH vs ER-MPH). Example of Equasym XL/Medikinet CR.

**tp** = transition probability, **SR** = suboptimal response, **OR** = optimal response, **DT** = discontinuing treatment, **NR** = natural remission, **IR-MPH** = immediate release methylphenidate, **ER-MPH** = extended release methylphenidate, **MPH-OROS** = methylphenidate osmotic release oral system.

## Figure B. Effect of the scenario analyses on the outcome of costs (€) (IR-MPH vs ER-MPH). Example of Equasym XL/Medikinet CR.

**tp** = transition probability, **SR** = suboptimal response, **OR** = optimal response, **DT** = discontinuing treatment, **NR** = natural remission, **IR-MPH** = immediate release methylphenidate, **ER-MPH** = extended release methylphenidate, **MPH-OROS** = methylphenidate osmotic release oral system.

## Figure C. Effect of the univariate sensitivity analyses on the outcome of effect (QALY) (IR-MPH vs ER-MPH). Example of Equasym XL/Medikinet CR.

**tp** = transition probability, **SR** = suboptimal response, **OR** = optimal response, **DT** = discontinuing treatment, **NR** = natural remission, **IR-MPH** = immediate release methylphenidate, **ER-MPH** = extended release methylphenidate, **MPH-OROS** = methylphenidate osmotic release oral system, **QALY** = Quality adjusted life years.

**Figure D.** Effect of the scenario analyses on the outcome of effect (QALY) (IR-MPH vs ER-MPH). Example of Equasym XL/Medikinet CR.

**tp** = transition probability, **SR** = suboptimal response, **OR** = optimal response, **DT** = discontinuing treatment, **NR** = natural remission, **IR-MPH** = immediate release methylphenidate, **ER-MPH** = extended release methylphenidate, **MPH-OROS** = methylphenidate osmotic release oral system, **QALY** = Quality adjusted life years.

## Figure E. Cost and effect differences of probabilistic sensitivity analysis in cost-effectiveness plane and 95% confidence interval (Example of Equasym XL/Medikinet CR).

ER-MPH = Extended release methylphenidate
MPH-OROS = Methylphenidate osmotic release oral system

QALY = Quality adjusted life years
